# Parent distress reactions following a serious illness or injury in their child: a protocol paper for the take a breath cohort study

**DOI:** 10.1186/s12888-015-0519-5

**Published:** 2015-07-08

**Authors:** Frank Muscara, Kylie Burke, Maria C McCarthy, Vicki A Anderson, Stephen JC Hearps, Simone J Hearps, Anica Dimovski, Jan M Nicholson

**Affiliations:** 1 Clinical Sciences, Murdoch Childrens Research Institute, The Royal Children’s Hospital, Flemington Road, Parkville, VIC 3052 Australia; 2 Parenting and Family Support Centre, School of Psychology, The University of Queensland, Brisbane, QLD 4072 Australia; 3 Judith Lumley Centre, La Trobe University, 215 Franklin St, Melbourne, VIC 3000 Australia

**Keywords:** Parents, Pediatric illness, Post-traumatic stress

## Abstract

**Background:**

Diagnosis of life threatening childhood illness or injury can lead to significant distress reactions in parents, with many experiencing clinically significant levels of post-traumatic stress symptoms. These symptoms can have long-term adverse impacts on parent mental health, family functioning, and the adjustment of the ill child. Independent studies have found such reactions in several different illness groups. However, very little research has systematically compared the prevalence, impact and trajectories over time of post-traumatic stress symptoms in parents across different childhood illness groups with an acute life threat. The current study seeks to map the course of post-traumatic stress reactions in parents of children with various life threatening illnesses over an 18 month period, and identify factors that predict successful adaptation in families.

**Method/Design:**

The current study described is of a prospective, longitudinal design. The sample included parents of children admitted to four major hospital departments at the Royal Children’s Hospital, Melbourne, Australia, for a life threatening illness or injury. Eligible parents were those who were caregivers of children aged 0-to 18-years admitted to the Oncology, Cardiology, Neurology and Pediatric Intensive Care Unit. Parents were recruited acutely, and completed self-report questionnaires at four time-points: within the first 4 weeks (T1:); then at 4 months (T2); 7 months (T3); and 19 months (T4) after admission. Questionnaires assessed parent and child mental health and wellbeing, and a number of risk and reliance factors such child illness factors, parent demographic factors, and psychosocial factors.

**Discussion:**

This study is one of the first to document the trajectory of post-traumatic stress responses in parents of very ill children, across illness groups. Given that it will also identify risk and resilience factors, and map the course of parent outcomes over an 18 monthperiod, it has the potential to inform novel strategies for intervention.

## Background

The experience of having a child diagnosed with an illness or injury that is potentially life-threatening or debilitating is highly distressing for parents. Parents of a child with a serious childhood illness or injury (SCII) must contend with the possibilities of their child’s death or lasting impairment, in the context of negotiating a path through complex diagnostic and treatment processes–an experience that can overwhelm even the most resilient parents [1]. Despite initial or recurrent periods of extreme distress, most parents of a child with a SCII are able to cope and adjust well over time [2–4]. However, some experience persistently elevated or escalating distress impacting on their functioning within the family unit [4–7], with adverse effects on themselves, their sick child and other family members. Little is known about the factors that determine which parents show spontaneous recovery in their psychological wellbeing and whether there are differences in recovery trajectories according to the type of illness or age of the child. This paper describes the research design and presents some initial descriptive data from a prospective longitudinal study, the Take a Breath Cohort Study, designed to determine the prevalence, trajectories and determinants of parent distress reactions for a cohort of Australian children (aged 0–18 years) recently diagnosed with a SCII. The study also seeks to investigate the impact of parent adjustment on the ill child.

Advances in public health and medical technology have resulted in dramatic reductions in infant and child mortality across the developed world, with concomitant changes in the leading causes of death and morbidity. In Australia, respiratory diseases, cancer, congenital conditions, injury and diseases of the nervous system are the leading causes of hospitalization and mortality in infancy and later childhood. The Royal Children’s Hospital (RCH) in Melbourne is one of Australia’s largest pediatric hospitals [8]. Annually, it receives over 2000 new admissions including more than 200 children diagnosed with cancer, 340 admitted due to serious injury, 120 requiring cardiac surgery at birth, and another 470 new admissions to intensive care. Having a child whose life or functional capacity is abruptly threatened by life threatening illness or injury is highly distressing and can lead to parental depression, acute stress or posttraumatic stress reactions [6, 9]. A medical traumatic stress model has been proposed as a helpful framework for understanding the impact of this experience on parents [10, 11].

At the time of commencement of this study, according to the Fourth Edition of the Diagnostic and Statistical Manual of Mental Disorders (DSM-IV) [12], experience of a traumatic event accompanied by the psychological symptoms of re-experiencing, avoidance, physiological arousal and associated functional impairment are features of Acute Stress Disorder (ASD; onset and duration between 2 days and 4 weeks after the traumatic event) and Posttraumatic Stress Disorder (PTSD; symptom persistence for at least 1 month). The Fourth Edition of the DSM listed parents’ experience of their child’s life-threatening illness as a traumatic event appropriate for these diagnoses. In contrast, the Fifth Edition [13] removed this as a qualifying stressor, emphasising the requirement of direct personal experience of trauma. Nevertheless, we contend that a child’s life-threatening illness does constitute a direct trauma to parents and that the parents’ experiences of witnessing their child undergo a series of unpleasant and sometimes painful treatments may constitute multiple stressors leading to significant traumatic stress responses. The reactions to these experiences, at least for a significant subsample of parents, are consistent with a trauma model.

Parent distress reactions are most common in the acute phase of the child’s illness [10, 14]. In a recent systematic review [15] we found that in the first 3 months post-diagnosis, rates of DSM-IV-defined ASD and PTSD ranged from 24 % to 40 % and 15 % to 25 %, respectively. These rates were reported for both mothers and fathers and across a number of illness groups, including parents of children hospitalized due to diagnosis of cancer, cardiac disease, type 1 diabetes, trauma or serious injury, burns, and other serious illnesses requiring admission to intensive care [1, 10, 16–18]. Higher levels of acute distress reactions have been shown to be predictive of later, persistent parent mental health difficulties [10, 14]. Individual studies have reported rates of PTSD 6–12 months after child diagnosis, affecting 5 % to 25 % of mothers and 5 % to 16 % of fathers [14, 19–21]. Recent studies also found that, in addition to PTSD, sub-threshold levels of posttraumatic stress symptoms (PTSS) affect a further 46 % of mothers and 28 % of fathers 6 months after their child’s cancer diagnosis [14], and 14 % of parents of children who suffered an accidental injury [4].

Clinically significant parent distress reactions can have far-reaching consequences, potentially impacting on the ill child and other family members. In the acute illness phase, symptoms can impair a parent’s ability to respond to the demands of their child’s illness [22, 23], to participate and adhere to treatment decisions [24], and can result in the utilisation of more hospital resources [25]. If persistent, this distress can have a significant impact on the parent’s ability to cope with daily tasks including the management of occupational demands and the care of other children [24]. Parental trauma, in combination with the general functioning of the family, has been found to predict the longer-term psychological, behavioral and general wellbeing of the ill child [7, 26–28]. In summary, early traumatic symptoms in parents can persist and evolve into serious, long-term mental health problems, with potentially chronic adverse effects on the family and the ill child [7, 27, 28].

### Conceptual models of parent trauma

**Fig. 1:**
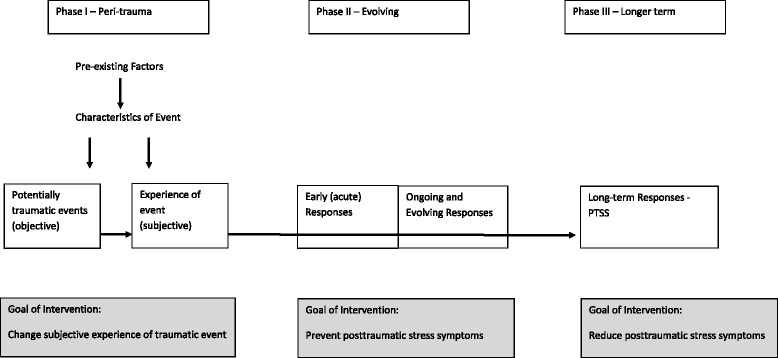
The Pediatric Medical Traumatic Stress Model for parents of ill children [29]

Despite the prevalence and serious consequences of parental distress reactions following a child’s illness or injury, relatively little is known about the typical course of these symptoms and the optimal opportunities for intervening. The Pediatric Medical Traumatic Stress Model (PMTS: Fig. 1) developed by Kazak and colleagues [29] conceptualizes parental trauma as progressing through three main phases corresponding to stages in the child’s illness/injury: 1) Peri-trauma–learning child is ill, beginning treatment; 2) Evolving–ongoing treatment, experience of treatment side effects and setbacks; and 3) Longer term–including remission, recovery or child death [29]. The timing and duration of these phases will vary according to the nature and course of the child’s illness. The model proposes that each stage entails exposure to a range of potentially traumatic events, which are linked to the individual parent’s appraisal of the events. The interaction between the events and the parent’s subjective perception of these, determines whether or not a traumatic response occurs. The model also indicates a role for pre-existing factors and the type of illness or injury in influencing the development of parental trauma, and proposes that each stage is associated with different approaches and targets for intervention.

Research has identified several factors that predict parent’s adjustment to their child’s illness or injury. These include demographic factors such as parent gender and ethnicity, with mothers more likely to experience traumatic stress than fathers [6, 21, 30] as are individuals from minority ethnic groups [30, 31]. Socio-economic factors are inconsistently related to parental adjustment [3, 6, 32]. Child age and gender have not been linked to later parent functioning [14, 32], and medical factors, such as illness severity, and the length of hospital stay have not generally been found to be associated with parental traumatic symptoms [3, 6, 14, 33]. In contrast, a number of psychosocial factors are consistent predictors of parental acute and posttraumatic stress symptomatology. Exposure to a prior trauma [14, 30], family functioning [6, 14, 31], short-and long-term psychological health of the ill/injured child [21, 34, 35], early parental distress levels [36], and parent perception of life threat to their child [7, 10, 20, 37] have all been reported to contribute to the levels of PTSS experienced my parents. Parent subjective appraisals of the illness, and the life threat to their child appear to be critical factors associated with parent distress responses [10, 20, 37]. These findings are consistent with cognitive models of trauma which emphasize the role of dysfunctional, subjective appraisals that individuals make about a traumatic event (in this case, child illness), rather than the objective characteristics of the trauma itself as being critical to posttraumatic adjustment [7, 38]. While previous research provides some indicators of the factors that influence individual vulnerability to trauma, it remains unclear whether these factors are consistent across illness groups and whether their influence changes as parents pass through different phases of their child’s illness.

**Fig. 2:**
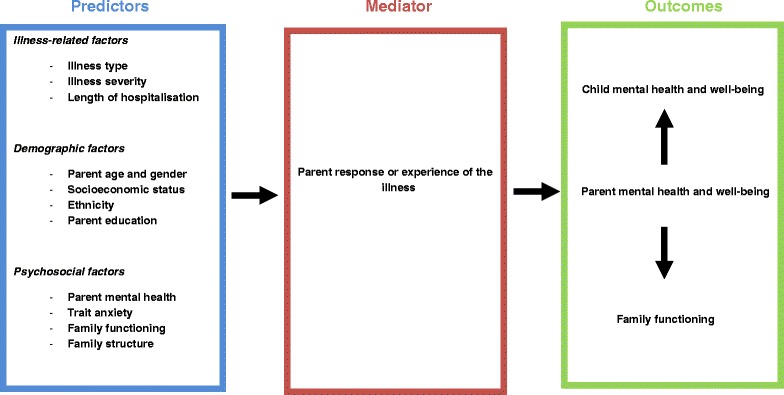
Proposed model of moderating factors influencing parent psychosocial distress following a SCII

The PMTS model together with available research provides a framework for understanding the trajectory of parent distress. In Fig. 2 we summarize the potential moderating factors influencing parent levels of distress, across time as the child passes through different phases of the illness. These fall into three broad risk categories: i) ‘*illness related factors’:* severity of the child’s illness, and type of illness; ii) *‘demographic factors’:* parent age and gender, socio-economic status, and ethnicity; and iii) ‘*psychosocial factors’:* current parent mental health, trait anxiety, family functioning, and family structure. We propose that these risk categories have the potential to influence the ‘*parent response to illness’*, at each phase of the child’s illness. In turn, the parent’s response to the illness acts as a mediator to determine parent adjustment and functioning. We also expect that parent functioning will secondarily influence family functioning and the adjustment of the ill child. Applying this model in a longitudinal study design will allow an in-depth and coherent approach to investigating parent adjustment across a range of serious childhood illnesses.

### The take a breath cohort study

Drawing on the conceptual models presented above, this prospective longitudinal study seeks to improve our understanding of parents’ psychosocial adjustment following their child’s diagnosis of a SCII. It will investigate the illness, demographic and psychosocial factors that predict different adjustment trajectories. It seeks to advance our understanding of similarities and differences in parent reactions across illness groups recruited within an acute hospital setting and enable the identification of factors that predict successful adaptation in families following diagnosis of a SCII. The main study aims are:

1. To investigate the prevalence of parent psychosocial distress in four illness groups: Pediatric diagnosis of cancer, a cardiac or neurological condition or admission to Pediatric Intensive Care Unit (PICU).
2. To determine the trajectory of parent psychosocial distress symptoms over an 18 month period from the child’s initial diagnosis of cancer, a cardiac or neurological condition or admission to PICU.
3. To identify the demographic, psychosocial and illness-related predictors of parent psychosocial distress and to investigate whether these vary at different time-points after the child’s initial diagnosis.
4. To examine the relationship between parent psychosocial distress and child psychological wellbeing from 4 to 19 months after the child’s initial diagnosis.

## Methods/Design

### Overall study design

The Take a Breath Cohort Study has a prospective longitudinal design and is conducted in accordance with STROBE guidelines. Recruitment commenced in November 2010, and continued until August 2012. Data will be collected at four time-points, selected to correspond to different phases of recovery in the PMTS model: within the first 4 weeks (T1:); 4 months (T2); 7 months (T3); and 19 months (T4) after admission. Specifically, T1 corresponds to the acute or ‘peri-trauma’ phase, T2 and T3 correspond to the ‘evolving’ phase, and T4 the ‘longer-term’ phase. Data collection will continue through 2014.

The study was approved by the Human Research Ethics Committee at the Royal Children’s Hospital, Melbourne (HREC 30044).

### Setting

Recruitment occurred within the Cardiology, Oncology, Neurology and PICU departments at the Royal Children’s Hospital (RCH), Melbourne. These departments were chosen for their high admission rates and to provide illness groups that were diverse in terms of the range of child ages at admission, and the nature of treatment received. Most importantly they were chosen due to the relatively instant onset of the illness or diagnosis, as well as the severity, with each having either a significant life threat, or threat to the integrity or future functioning or development of the child. For example, children in Cardiology are infants born with a congenital defect, who require significant surgical intervention early in life. In contrast, childhood cancer diagnoses occur at all ages, with the diagnosis often unexpected and highly distressing, and the chances of relapse remaining high for several years. The Neurology and PICU groups are varied in the duration and nature of treatments received, however only admissions that were sudden and unexpected, and which have the potential to have lasting functional impairments requiring rehabilitation and altered life expectations were included.

### Participants

#### Inclusion criteria

Eligible parents were those who were caregivers of children aged 0-to 18-years admitted to the RCH for the first time for cardiac surgery in the first month of life (Cardiology), a new cancer diagnosis of any type (Oncology), a stroke or moderate-to-severe head injury (Neurology), or admission to PICU for longer than 48 h and their first admission for that illness (PICU).

Ill children who were aged 7-to 18-years were invited to participate in data collection during time-points 2, 3 and 4 of the project. They were not involved in time-point 1. Parents were still able to participate even if their child did not.

#### Exclusion criteria

Parents were excluded if they were aged below 18-years of age, had experienced a major trauma in the 2 months prior to their child’s diagnosis (such as the death or serious injury of another immediate family member), or had insufficient English to complete the questionnaires. Parents of children not expected to live longer than 6 months were identified by the clinical team and were not approached for participation.

### Procedure

Given the complexities of conducting clinical research within an acute hospital setting, prior to commencing the study, all medical and allied health staff within the relevant hospital departments were informed of the study, and provided feedback regarding potential recruitment strategies within their departments. Advisory groups were established, comprising the research team and key stakeholders and clinicians within each hospital department, who met frequently to ensure ongoing support for the study from the clinical teams and departments. As a result of this early and continuing work, the research team had good buy in, support and trust from the clinical teams who assisted with identification and recruitment of eligible families.

### Parent recruitment

Recruitment occurred at a time of turmoil for these families. Recruitment and baseline data collection therefore required sensitivity and flexibility to ensure that this did not interfere with clinical services or overburden families. As a result, the research team worked closely with the clinical staff within each inpatient department.

In each department, nurse co-ordinators reviewed new admissions for eligibility. The nurse co-ordinator or social worker provided eligible parents with a brief description of the study, and sought verbal consent to pass on contact details to the research team. Parents were then recontacted by a member of the research team to seek participation in the study, with consenting parents signing a written consent form. At each stage of contact, parents who declined were asked their reasons for non-participation, consistent with the STROBE guidelines [39]. Baseline data were collected via completed parent questionnaires. For the majority of parents, these were distributed and completed within the hospital. For those already discharged, parents were mailed out with a reply paid return envelope. Reminder calls were made if the questionnaires were not returned within 2 weeks, and if the questionnaire was not returned within 4 weeks, the data were considered missing.

### Child recruitment

From time-point 2, with parent permission, a research team member invited children (aged 7–18 years) who were competent and able to complete the assessments to participate in the study. The parent decided whether to ask their child if they would like to participate. If the child chose not to participate the research team ceased contact with the child, however parents were still able to continue their own participation irrespective of child involvement. Once a signed consent form was received from the child/caregiver, a member of the research team provided the family with parent and child questionnaires relating to the relevant data time-point. Parents and children were able to withdraw permission to participate at any time and to have any information about them destroyed.

### Longitudinal follow-ups at 4, 7, and 19 months

Two weeks prior to the relevant time-point, a reminder message is sent to the parent to inform them that the next questionnaire will be arriving in the mail shortly. The message will be communicated via email, SMS, or telephone call, depending on the parents’ preferred method of contact. At the appropriate time, the relevant questionnaires will be mailed out, with a reply paid envelope. Reminder calls are made if the questionnaire is not returned within 2 weeks, and if the questionnaire is not returned within 4 weeks, the data will be considered missing. Parents and children will be considered a ‘drop out’ if they ask to withdraw from the study, or if they fail to return questionnaires at two successive time-points in the study.

### Measures

Measures, data sources and time-points are summarized in Table 1. Measures were selected to assess different aspects of the conceptual model, specifically parent and child mental health and wellbeing (quality of life, PTSD symptoms), child illness factors (severity of illness, type of illness), parent demographic factors (age, gender, income, ethnicity, education), and psychosocial factors (family functioning, family structure, symptoms of ASD, depression and anxiety, psychosocial risk factors). Due to the wide age range of the children within the sample, different measures assessing similar constructs were chosen for children at different ages/developmental stages. All are reliable and validated measures, except the demographic and health economy questionnaire, which comprises general questions designed by the research team. Outcomes will be assessed at four time-points at baseline (T1: within the first 4 weeks since diagnosis), and 3 follow ups: at 4 months (T2), 7 months (T3) and 19 months post diagnosis (T4). Details regarding the patients’ illness including diagnosis, date of diagnosis, number of visits to the Emergency Department and number of days of admission will be obtained from departmental/hospital databases. Patient diagnoses will be described.

**Table 1:** Summary of the measures included in the take a breath cohort study

Construct	Measure	Source	Time-point			
			1	2	3	4
*Outcome Measures*						
Parent Distress	Posttraumatic Stress Checklist-Specific Version (PCL-S) [40]	P		*	*	*
Post traumatic growth	Post Traumatic Growth Inventory – Short form [41]	P				*
Child psychopathology	The Brief Infant Toddler Social Emotional Assessment [42], or Strengths and Difficulties Questionnaire (SDQ) [43]	P		*	*	*
	Strengths and Difficulties Questionnaire (SDQ) [43]	C		*	*	*
	Parent Report of Posttraumatic Stress Symptoms [44]	P	*	*	*	*
	Child Report of Posttraumatic Stress Symptoms [44]	C		*	*	*
Child wellbeing	PEDS Quality of Life (6 years+) [45] or TNO-AZL Preschool Children Quality of Life (1–5 years) [46]	P		*	*	*
	PEDS Quality of Life [45]	C		*	*	*
*Illness Related Factors*						
Illness variables	Severity of Illness Scale [47]	MD	*			
*Demographic Factors*						
Demographics	General questionnaire of parent demographic information (eg. age, years of education, ethnicity)	P	*			
Health Economy	General questionnaire of health economy factors (eg. level of income, services used in the hospital and in the community)	P	*	*	*	*
*Psychosocial Factors*						
Psychosocial factors	Psychosocial Assessment Tool (PAT 2.0) [48] (assessing psychosocial risk factors such as family structure, family beliefs, access to services and transport)	P	*	*	*	*
	Psychosocial Assessment Tool (PAT-S) [25]	SW	*	*		
Parent distress/ wellbeing	Acute Stress Disorder Scale (ASDS) [49]	P	*			
	Depression Anxiety Stress Scale Short Form (DASS-21) [50]	P	*	*	*	*
	Assessment of QoL (AQOL) [51]	P	*	*	*	*
	State Trait Anxiety Scale [52]	P	*			
Family Functioning	Family Environment Scales [53]	P	*			
*Moderators*						
Experience of Illness	Parent Experience of Child Illness (PECI) [54]	P		*	*	*
	Family Management Measure [55]	P		*	*	*
	Benefit Burden Scale - Children [56]	C		*	*	*

* = measure administered at this time point, P = Parent reported measures, MD = Doctor reported measures, SW = Social Worker reported measures, C = Child reported measures, Timepoint 1 = acute (within first month since hospitalization/diagnosis), Timepoint 2 = three months after Timepoint 1 (four months since hospitalization/diagnosis), Timepoint 3 = six months after Timepoint 1 (seven months since hospitalization/diagnosis), Timepoint 4 = 18 months after Timepoint 1 (19 months since hospitalization/diagnosis)

### Sample and baseline descriptive data

**Fig. 3:**
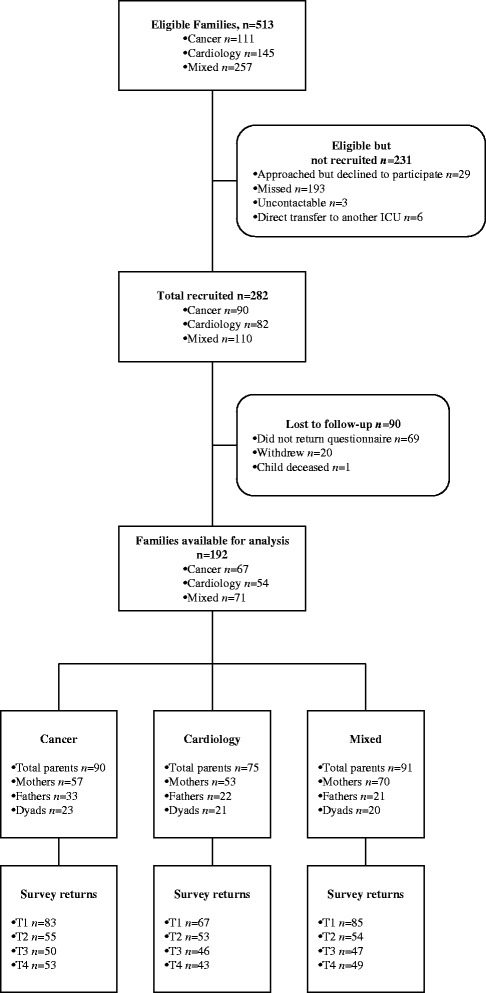
Recruitment flow diagram for the Take a Breath Cohort Study, across the three illness groups

The sample consists of 256 parents of 192 children. Of the 256 parents, 70.3 % (180) are mothers, and there are 64 couples. This represents 37.4 % of 192 eligible families admitted over a 21 month period. In total, 68.1 % of eligible families that were approached consented to participate in the study. A sample much smaller than expected and required was obtained within the Neurology group. Given that many within this group were initially treated within PICU, it was decided to collapse the Neurology and PICU groups into a combined ‘Mixed Illness’ group to increase power in future analyses. A detailed breakdown of recruitment within each illness group is provided below, and the recruitment flow diagram within each of the three illness groups can be seen in Fig. 3.

Within the Mixed Department sample, parents of 257 children admitted into PICU/Neurology Departments within the recruitment period were eligible for the study. Of those, 147 (57.2 %) were not recruited for various reasons, with the majority not able to be approached. Of the remaining 110 that were recruited, data from 71 families (64.5 %) were obtained and available for data analysis.

Within the cardiology sample, parents of 145 children admitted into the Cardiology Department within the recruitment period were eligible for the study. Of those, 63 (43.4 %) were not recruited for various reasons, with the majority not able to be approached. Of the remaining 82 that were recruited, data from 54 families (65.9 %) were obtained and available for data analysis.

Within the oncology sample, parents of 111 children admitted into the Oncology Department within the recruitment period were eligible for the study. Of those, 21 (18.9 %) were not recruited for various reasons, with the majority not able to be approached. Of the remaining 90 that were recruited, data from 67 families (74.4 %) were obtained and available for data analysis.

Tables 2 and 3 display parent and child demographic information at recruitment, stratified by illness group. There were no significant differences in parent gender, education, country of birth, and relationship status across the illness groups (all p >0.05). As expected, given the Cardiology illness group was restricted to infants, parents and children recruited from this illness group were significantly younger than those from the Oncology group (*p* = 0.005 and *p* <0.001, respectively). Moreover, length of stay was significantly longer for the Cardiology group (*p* = 0.008), which was also expected.

**Table 2:** Parent and child demographics at Time 1

	Oncology		Cardio		Mixed	
	(*n* = 90)		(*n* = 75)		(*n* = 91)	
	M	(SD)	M	(SD)	M	(SD)
Parent Age (years)	38.7	(8.5)	34.7	(8.2)	36.0	(7.5)
	n	(%)	n	(%)	n	(%)
Gender						
Male	33	(36.7)	22	(29.3)	21	(23.1)
Country of Birth						
Australia	77	(85.6)	60	(80.0)	79	(86.8)
Education						
Less than High School	9	(10.0)	1	(1.3)	10	(11.0)
Graduated High School	11	(12.2)	8	(10.7)	10	(11.0)
Some University/TAFE	19	(21.1)	19	(25.3)	21	(23.1)
Graduated University/TAFE	31	(34.4)	26	(34.7)	32	(35.2)
Some Postgraduate Study	5	(5.6)	2	(2.7)	0	(0.0)
Postgraduate Qualification	15	(16.7)	18	(24.0)	17	(18.7)
Relationship Status						
Single	3	(3.3)	3	(4.0)	5	(5.5)
Married/Partnered	81	(90.0)	72	(96.0)	76	(83.5)
Separated/Divorced	3	(3.3)	0	(0.0)	5	(5.5)
Repartnered	3	(3.3)	0	(0.0)	4	(4.4)
Survey returns						
Time 1	83	(92.2)	67	(89.3)	85	(93.4)
Time 2	55	(61.1)	53	(70.7)	54	(59.3)
Time 3	50	(55.6)	46	(61.3)	47	(51.6)
Time 4	53	(58.9)	43	(57.3)	49	(53.8)

**Table 3:** Child demographic and illness variables at time 1

	Oncology		Cardio		Mixed	
	(*n* = 67)		(*n* = 54)		(*n* = 71)	
	M	(SD)	M	(SD)	M	(SD)
Child Age (years)	6.0	(4.6)	0.1	(0.1)	3.6	(4.8)
Length of Stay (days)	16.3	(9.5)	21.1	(7.7)	16.2	(11.1)
	n	(%)	n	(%)	n	(%)
Gender						
Male	40	(59.7)	32	(59.3)	43	(60.6)
Country of Birth						
Australia	64	(95.5)	54	(100.0)	68	(95.8)

The current Victorian Privacy Act prohibits the collection of information about non-consenting families, precluding a comparison of participating and non-participating families. Hence a detailed exploration regarding the representativeness of the sample is not possible. Main reasons given by parents for declining participation included that they had no time, or were currently too overwhelmed with managing their child’s condition to participate. The main reasons that parents were not approached were because they were discharged prior to being contacted by a member of the research team, or were not contactable.

### Cohort retention and tracking

Project participants will be tracked in accordance with the STROBE guidelines [39]. This method enables detailed recording of participants across time. As such, specific information was collected on both eligible and ineligible families (in accordance with the Victorian Privacy Act), as well as families that refused to participate. Information was collected regarding reasons for ineligibility/refusal and the number of families that were ineligible and refused participation. No identifying information was collected on these families. There was no significant difference between group retention for each time point (all p >0.05). Participant tracking data is stored on a password protected database at the RCH. A Microsoft Access database assists in monitoring all eligible participants, and stores details regarding those participating in the study, and reasons for withdrawal or decline for those not participating. The database provides weekly updates as to which families required reminders for follow up questionnaires at all time-points. The database is password and firewall protected, so that only members of the research team have access to the information.

### Data analysis

For Aim 1, descriptive statistics will be calculated for outcome and predictor variables. These initial analyses will establish the prevalence of acute stress symptoms and PTSS of parents and children with SCII across the 4 time points, from acute to 19 months post admission/diagnosis. The impact of potential confounds (such as parent gender and child age) on outcomes will be explored in univariate analyses. Should putative confounds be significant (at *p* <0.1), they will be adjusted for in further analyses.

For Aim 2, Repeated Measures ANOVA will be used to assess temporal changes in parent traumatic stress symptoms, with time since diagnosis as the independent variable and the PCL-S as the outcome measure. Random effects linear regression may also be employed, as it allows for correlations between repeated measures taken from the same participant, and analyses available data (allowing missing timepoints). This procedure will be repeated for the child measures with the CROPS as the outcome measure. Power analysis suggests that in order to detect a small-to-medium effect size (η^2^ = 0.025) the target of 240 families (80 in each illness group) is sufficient to conduct the planned analyses (power = 80 %, α = 0.05).

For Aim 3, predictor variables will be assessed for collinearity and each predictor will be assessed using a univariate model. Predictors significant at p <0.1 will be used to fit a multiple linear regression model, to determine independent predictors for each outcome. Predictor variables will include demographic, psychosocial and illness-related factors. Simple linear regression will then be used to examine whether parental distress at diagnosis (measured using ASDS) predicts child traumatic stress symptoms at 19 months post diagnosis. Given the sample size of 240, we will be able to detect small-to-medium effect sizes (η^2^ = 0.065) in multiple regression model with an estimated 10 independent variables, and small effect size (η^2^ = 0.030) for the latter simple regression.

For Aim 4, to further investigate risk and resilience factors for acute stress and PTSS symptoms in children with SCII and their parents, path analysis techniques will be explored, based on Kazak’s model of medical traumatic stress, which includes peri-trauma factors (baseline child/parent functioning), Evolving factors (such as the acute and later distress response), and long-term outcomes (child and parent quality of life, family function, parenting, psychopathology).

## Discussion

Despite evidence suggesting that rates of PTSD in parents decline over time, a significant proportion of parents continue to suffer clinically significant levels of distress in the long-term. It remains difficult to characterise the trajectory of parent distress over time for a number of reasons. Past research has mainly examined a single illness group, and many studies use different scoring tools and methods, making it difficult to determine trajectories over time, the predictors of functioning at different phases of the model, and whether illness factors or the type of illness contributes to different outcomes [15]. The Take a Breath Cohort Study seeks to determine how significant this problem is across different illness groups, and the extent to which there is spontaneous resolution of symptoms, requiring no further intervention, or to what extent early intervention is warranted.

The link between parent psychological distress and serious childhood illness has significant implications for pediatric healthcare services. This study will address a number of important knowledge gaps that currently limit the extent to which effective psychosocial support services can be directed towards those parents who would benefit most. A greater understanding of parent distress reactions and their impact will also assist in the allocation of resources to address this problem, with those potential resources ranging from basic psycho-education, to more involved psychological approaches (e.g. interventions based on cognitive behavioural therapy or acceptance and commitment therapy), to involvement with psychiatry. Further, the findings will inform the development of interventions to support parents at risk of long-term mental health issues as a result of their child’s admission. If similar distress responses and trajectories are identified across illness groups, this could inform a hospital-wide approach to managing the traumatic stress symptoms of parents, and in turn, facilitate the introduction of evidence-based, generalizable interventions, which are currently sparse. Given that parents provide the primary emotional support and care for their ill or injured child, support for parents following diagnosis of SCII is critical. Greater understanding and research in this area has been called for by a number of research groups internationally, with Kazak and colleagues [2] calling for an approach that “can provide a map for treatments that are preventative, innovative, and targeted to the true needs of the child, family, and healthcare system” (pp. 1100).

### Ethical approval

Ethical approval has been obtained from the Royal Children’s Hospital Human Research Ethics Committee (HREC 30044).

## Abbreviations

AQOL: Assessment of QoL
ASD: Acute stress disorder
ASDS: Acute stress disorder scale
DASS-21: Depression anxiety stress scale short form
DSM: The diagnostic and statistical manual of mental disorders
PAT: Psychosocial assessment tool
PCLC: Posttraumatic stress checklist-Specific version
PECI: Parent experience of child illness
PICU: Pediatric intensive care unit
PMTS: Pediatric medical traumatic stress model
PTSD: Post traumatic stress disorder
PTSS: Post traumatic stress symptoms
RCH: The royal children’s hospital
SDQ: Strengths and difficulties questionnaire
SCII: Serious childhood illness or injury
T1: Time-point 1
T2: Time-point 2
T3: Time-point 3
T4: Time-point 4
